# Development and cytological characterization of wheat–*Thinopyrum intermedium* translocation lines with novel stripe rust resistance gene

**DOI:** 10.3389/fpls.2023.1135321

**Published:** 2023-02-24

**Authors:** Xianrui Guo, Yuhong Huang, Jing Wang, Shulan Fu, Chunhui Wang, Mian Wang, Chen Zhou, Xiaojun Hu, Tao Wang, Wuyun Yang, Fangpu Han

**Affiliations:** ^1^ Laboratory of Plant Chromosome Biology and Genomic Breeding, School of Life Sciences, Linyi University, Linyi, China; ^2^ State Key Laboratory of Plant Cell and Chromosome Engineering, Institute of Genetics and Developmental Biology, Innovation Academy for Seed Design, Chinese Academy of Sciences, Beijing, China; ^3^ Chengdu Institute of Biology, Chinese Academy of Sciences, Chengdu, China; ^4^ Crop Research Institute, Sichuan Academy of Agricultural Science, Chengdu, China

**Keywords:** wheat, *Thinopyrum intermedium*, translocation line, stripe rust, gene mapping

## Abstract

Wheat stripe rust is a destructive disease in many cool and temperate regions around the world. Exploiting novel sources of resistance can provide wheat cultivars with robust and durable resistance to stripe rust. The wheat–*Thinopyrum intermedium* addition line TAI-14 was proven to carry a stripe rust resistance gene (named as *YrT14*) on the alien *Th. intermedium* chromosome. In order to transfer the resistance gene to wheat, wheat–*Th. intermedium* translocation lines were created by irradiating the pollen of the line TAI-14. We totally obtained 153 wheat–*Th. intermedium* translocation lines, among which the long alien segmental translocation line Zhongke 78 and the intercalary translocation line Zhongke 15 not only showed good integrated agronomic traits but also were identified as highly resistant to stripe rust in both seedling and adult plant stages. The alien chromatin in Zhongke 15 was identified as an insertion into the satellite of chromosome 6B, a type of translocation never reported before in chromosome engineering. By screening Simple Sequence Repeat (SSR) and Expressed Sequence Tag (EST) markers as well as the markers developed from RNA-sequencing (RNA-Seq) data, 14 markers were identified specific for the alien chromosome and a physical map was constructed. Both Zhongke 78 and Zhongke 15 could be used as a novel source of stripe rust resistance for wheat breeding, and the linked marker T14K50 can be used for molecular marker–assisted breeding. Finally, based on the karyotype, reaction to stripe rust, and genome resequencing data of different wheat–*Th. intermedium* translocation lines, the stripe rust resistance gene *YrT14* was located to an 88.1 Mb interval from 636.7 to 724.8 Mb on *Th. intermedium* chromosome 19 corresponding to 7J or 7J^s^.

## Introduction

As one of the most important food crops, common wheat (*Triticum aestivum*) is cultivated on approximately 220 million ha around the world and feeds more than one-third of the worlds’ population (http://www.fao.org/faostat). In the pursuit of high yield in breeding, the diversity of crops is becoming narrower and narrower, which, in turn, diminishes their potential to survive in both abiotic and biotic stresses (Allard, 1996; Hoisington et al., 1999; Charmet, 2011). Wheat stripe rust, powdery mildew, and Fusarium head blight are three fungal diseases that seriously threaten wheat production globally (Bennett, 1984; Bai and Shaner, 1994; Kolmer, 2005). It is of great importance to exploit novel resistant sources to provide wheat with robust and durable resistance against these diseases.

As a perennial grass, *Thinopyrum intermedium* is identified as an allohexaploid. Although chromosome composition is controversial, it is generally regarded as JJJ^s^J^s^StSt (Chen et al., 1998b). The subgenome St is closely related to the genome of *Pseudoroegneria strigose*, and the J or J^S^ subgenome is highly homologous with the genome E^e^ of *Th. elongatum* and E^b^ of *Th. bessarabicum* (Liu and Wang, 1993; Chen, 2005). These results were also supported by the fact that researchers found that one set of chromosomes was labeled as the St type while the rest were all labeled as the non-St type by employing the probe St_2_-80 to detect the chromosomes in *Th. intermedium* (Wang et al., 2017). A consensus map with 21 linkage groups containing 10,029 markers was produced by using genotyping-by-sequencing (GBS). Through mapping the sequence tags from *Th. intermedium* to the diploid barley reference genome, high collinearity and synteny between the three homoeologous *Th. intermedium* chromosomes and each of the seven barley chromosomes were discovered (Kantarski et al., 2017). Referring to the consensus map and a set of 350,885 75-mer markers, the assembled scaffolds of *Th. intermedium* were oriented, ordered, and joined together to form chromosomes (http://phytozome.jgi.doe.gov/). This released genomic resource would accelerate the development of markers and gene cloning.

*Th. intermedium* has been used as a source for wheat improvement for decades. As *Th. intermedium* can easily be crossed with wheat, numerous useful genes and traits have been transferred into wheat in the form of partial amphiploids, addition lines, substitution lines, and translocation lines. Wheat–*Th. intermedium* partial amphiploids containing a complete set of wheat and a set of *Th. intermedium* chromosomes have been reported, such as Zhong1 to Zhong5, TAF46, TE261-1, TE266-1, TE346-1, TAI8355, and 363-1-21 (Chen et al., 1998a; Chen et al., 1999; Chen et al., 2003; Han et al., 2004; Chang et al., 2010; Guo et al., 2015; Cui et al., 2021). Partial amphiploids were usually unstable in cytology and conserved many grass characteristics that were undesirable in breeding exercise. Thus, a great number of addition and substitution lines were produced by crossing the partial amphiploids with wheat. He et al. (1989) developed two sets of wheat–*Th. intermedium* addition lines TAI-11~17 and TAI-21~27 by crossing the wheat–*Th. intermedium* partial amphiploid Zhong series lines with common wheat. The resulting progeny lines of TAI-12 and TAI-15 presented longer seeds and spikes than control (He et al., 1989). In addition, another three progeny lines TAI-23, TAI-25, and TAI-27 were identified as resistant to stem rust, leaf rust, and the wheat yellow dwarf virus, respectively (He et al., 1989). New high- molecular-weight subunits were identified in three wheat–*Th. intermedium* addition lines W210, W211, and W212 that were derived from the hybrids of common wheat Yannong 15 and *Th. intermedium* H-2 (Wang and Wang, 2016). In addition to wheat–*Th. intermedium* addition lines, some wheat–*Th. intermedium* substitution lines were produced, such as the 1St#2 (1D) substitution line AS1677 showing higher kernel protein content and the 4Ai#2(4A) substitution line CI 15092 with eyespot resistance (Li et al., 2005; Li et al., 2016). Based on these intermediate materials, many wheat–*Thinopyrum* translocation lines derived from centric breakage–fusion, recombination between homoeologous chromosomes, or physical irradiation were reported by different groups. For example, a new compensating wheat–*Th. intermedium* Robertsonian translocation line CH13-21 with resistance to powdery mildew and stripe rust was derived from a spontaneous centric breakage fusion (Zhan et al., 2015). Relying on *ph1b*-induced homoeologous recombination, a shortened *Th. intermedium* segment containing the *Wsm3* gene was translocated to chromosome 7B and conferred wheat resistance to the wheat streak mosaic virus (Danilova et al., 2017). In addition, P961341, a wheat–*Th. intermedium* translocation line with resistance to the yellow dwarf virus was produced by irradiating with γ rays from ^60^Co (Ohm et al., 2005).

Isozymes Cpxe-Ag^i^2 and Est-Ag^i^3 whose homoeologs in wheat were located on 7AS and 7BS, respectively, were discovered in the addition line TAI-14, which indicated that the alien chromosome in TAI-14 was homoeologous with wheat group-7 chromosomes (Benito et al., 1987; Gao and Hao, 1993; Vladova and Petkolicheva, 1996). In this study, the wheat–*Th. intermedium* addition line TAI-14 was identified as resistant to stripe rust. Genetics analysis revealed that there was a stripe rust resistance gene (named as *YrT14*) on the alien *Th. intermedium* chromosome. In order to transfer the resistance gene to wheat, a series of wheat–*Th. intermedium* translocation lines were developed, among which the long alien segmental translocation line Zhongke 78 and the intercalary translocation line Zhongke 15 showed good integrated agronomic traits and resistance to stripe rust and could be used as a novel resource for wheat stripe rust breeding. By screening SSR and EST markers as well as the markers developed from RNA-Seq data, we developed specific markers and constructed a physical map for the alien chromosome according to the size of the alien chromatin in different wheat–*Th. intermedium* translocation lines. Finally, by combining the karyotype, reaction to stripe rust, and genome resequencing data of different wheat–*Th. intermedium* translocation lines, the stripe rust resistance gene *YrT14* was located to an 88.1 Mb interval between 636.5 and 724.8 Mb on *Th. intermedium* chromosome 19 that was proven to correspond to chromosome 7J or 7J^s^.

## Materials and methods

### Plant materials

TAI-14 was derived from the wheat–*Th. intermedium* partial amphiploid Zhong2 (He et al., 1989). It is a disomic addition line, containing a whole set of 42 wheat chromosomes and a pair of chromosomes derived from *Th. intermedium* (He et al., 1989; Han et al., 1998). We referred to the plant carrying two alien chromosomes as T14-44, the one carrying one alien chromosome as T14-43 and the one without an alien chromosome as T14-42, respectively. The wheat cultivar Jimai 22 was used as the recurrent parent to cross with the translocation line. Wheat cultivars Fielder and Jimai 20 were susceptible to stripe rust and used as parents to generate F_2_ populations. Two highly stripe rust susceptible wheat varieties Mingxian 169 and Mianyang 11 were used as control in strip rust resistance evaluation. *Th. intermedium* accession PI 440001, *T. urartu* accession TMU38, and *Aegilops tauschii* accession TQ27 were used for the preparation of genomic DNA probes. *T. asetivum* line Chinese Spring (CS) and *Ae. speltoides* accession AE739 were used for the preparation of blocking DNA.

### Induction and improvement of wheat–*Th. intermedium* translocation lines

At the flowering stage, the spikes of the wheat–*Th. intermedium* addition line TAI-14 were cut from the plant in the morning, and immediately radiated by γ rays derived from ^60^Co with a dose of 18 Gy. Then the fresh pollens were used to pollinate the wheat variety Jimai 22 with its stamens removed in advance. The hybrid seeds were harvested at maturity. The translocation lines were identified by utilizing genomic *in situ* hybridization (GISH). Using Jimai 22 as the recurrent parent, the integrated agronomic traits of the translocation lines were improved by continuous backcrossing.

### Fluorescence *in situ* hybridization and genomic *in situ* hybridization

Seeds were germinated on moist filter paper in a Petri dish at room temperature for 2–3 days. Growing roots were cut from seedlings and treated in nitrous oxide with 15 bars of pressure for approximately 2 h. The roots were subsequently fixed in 90% acetic acid for 5 min and then stored in 70% v/v ethanol at -20°C. Chromosome spread preparation was performed as previously described (Kato et al., 2004).

Oligo-pSc119.2-1 combined with Oligo-pTa535-1 was used to distinguish the whole set of 42 wheat chromosomes (Tang et al., 2014). Oligo-pSc119.2-1 (10 ng/µl) and Oligo-pTa535-1 (10 ng/µl) were 5’ end-labeled with 6-carboxyfluorescein (6-FAM) and 6-carboxytetramethylrhodamine (6-Tamra) (Invitrogen^TM^, Shanghai, China), respectively. Genomic DNA was isolated from the leaves of *Th. intermedium* accession PI 440001, *T. urartu* accession TMU38, *Ae. speltoides* accession AE739, *Ae. tauschii* accession TQ27, and CS using the cetyltrimethylammonium bromide (CTAB) method (Murray and Thompson, 1980). The green or red probes with a concentration of 100 ng/µl were prepared according to the nick translation method (Kato et al., 2011). The genomic DNA of *Th. intermedium*, *T. urartu* and the plasmid of St_2_-80 reported by Wang et al. (2017) were labeled with Alexa Fluor-488-5-2'-deoxyuridine 5'-triphosphate (dUTP) (Invitrogen^TM^, Shanghai, China). The genomic DNA of *A. tauschii* and the centromeric retrotransposon of wheat (CRW) clone 6C6 was labeled with Texas-red-5-dCTP (Invitrogen^TM^, Shanghai, China). The genomic DNA of CS and *A. speltoides* in a concentration of 3,000 ng/µl was used for blocking in multicolor-GISH (mc-GISH). For each slide, FISH was performed in 10 µl reaction volumes, in which 0.2 µl Oligo-pSc119.2-1, 0.2 µl Oligo-pTa535-1, and 0.3 µl 6C6, 0.5 µl St_2_-80 were used and the 2x SSC, 1x TE buffer was used to adjust the volume. For *Th. Intermedium* chromatin detection, 10 µl reaction volumes for each slide contain 0.5 µl labeled genomic DNA of PI 440001 and 2.5 µl genomic DNA of CS. For the mc-GISH on wheat, the 10 µl reaction volumes for each slide contain the 2 µl labeled genomic DNA of TMU38, 2 µl genomic DNA of AE739, and 1 µl labeled genomic DNA of TQ27. All chromosomes were counterstained with 4, 6-diamidino-2-phenylindole (DAPI) (Vectashield, Vector Laboratories, Burlingame, CA, USA). Chromosomes on microscope slides were examined using a BX61 fluorescence microscope (Olympus, Tokyo, Japan) equipped with a U-CMAD3 camera (Olympus, Tokyo, Japan) and appropriate filter sets. The signal capture and picture processing were performed using MetaMorph software (Molecular Devices, LLC., San Jose, CA, USA). The final image adjustment was done in Adobe Photoshop CS5 (Adobe Systems Incorporated, San Jose, CA, USA).

### Developing specific makers and constructing physical map for the alien chromosome

In order to get specific markers for the alien chromosome, we screened 197 wheat group-7-specific microsatellite markers reported by Somers et al. (2004) and 88 pairs of sequence-tagged sites-polymerase chain reaction (STS-PCR) primers on wheat group-7 chromosomes (**Supplementary Table S1**). At the two-leaf stage, 27 plants of T14-44 and 25 plants of T14-42 were collected and separately pooled for RNA isolation using a TRIzol reagent (Invitrogen^TM^, Shanghai, China), followed by the treatment with DNase I (Invitrogen^TM^, Shanghai, China). The samples were sequenced using the Illumina Hiseq2500 platform (Berry Genomics, Beijing, China) to generate 125 bp pair-end reads. The *de novo* assembly of clean reads was performed by using the software Trinity 2.1.1 (Haas et al., 2013). The expression level was calculated by mapping reads to the assembled transcripts employing Trinity scripts, RSEM, and edgeR (Haas et al., 2013). The TransDecoder software package (https://sourceforge.net/projects/transdecoder/) was used to predict the coding region for these transcripts. The transcripts were annotated in the Swiss-Prot database using Blastx. The transcripts expressed in T14-44 but not in T14-42 were extracted. Then, the transcripts annotated as Nucleotide Binding Site–Leucine Rich Repeat (NBS-LRR) protein and protein kinases were used to design primers using the software Primer 5.0 (PREMIER Biosoft, San Francisco, CA, USA).

The conditions of the polymerase chain reaction (PCR) were as follows: initial denaturation at 94°C for 4 min, followed by 35 cycles of 30 s at 94°C, 30 s for annealing at 55°C–60°C, 1 min for extension at 72°C, and a final extension at 72°C for 10 min. Amplified PCR products were separated on 8% non-denaturing polyacrylamide gels stained with silver at 200 V for 1 h and 1.5% agarose gels stained with ethidium bromide at 150 V for approximately 25 min. The D2000 Plus DNA Ladder (GenStar, Beijing, China) and the 100 bp DNA Ladder (TianGen Biotech Co, Beijing, China) were used for the DNA marker in non-denaturing polyacrylamide gel and agarose gel electrophoresis, respectively.

### Genome resequencing and read depth analysis on wheat–*Th. intermedium* translocation lines

The genome of wheat–*Th. intermedium* translocation lines Zhongke 15, Zhongke 353, Zhongke 215, Zhongke 16, and Zhongke 55 was resequenced. Genomic DNA for resequencing was isolated by using the CTAB method. A combined reference genome consisted of CS reference genome v1.0 (Consortium (IWGSC) et al., 2018) and three genomic sequences of Chr19-21 extracted from *Th. intermedium* reference genome v2.1 (http://phytozome.jgi.doe.gov/) were used. Raw reads were first processed with Trimmomatic version 0.36 (with parameters: SLIDINGWINDOW:5:20 LEADING:5 TRAILING:5 MINLEN:25) (Bolger et al., 2014). Cleaned reads were aligned to the combined reference genome with Burrows-Wheeler-Alignment (BWA) mem version 0.7.13-r1126 (Li and Durbin, 2009). Alignments were compressed, sorted, filtered (remove mapping quality less than 20), and indexed by using SAMtools version 1.9 (https://sourceforge.net/projects/samtools/files/samtools/). Duplicate alignments were removed with Picard version 2.25.6 (https://broadinstitute.github.io/picard/). The means of read depth within per 100 kb window were calculated using Mosdepth version 0.2.6 with default parameters (Pedersen and Quinlan, 2018). The chromosome translocation points were identified in Integrative Genomics Viewer (IGV) version 2.12.0 (https://software.broadinstitute.org/software/igv/).

### Phenotype evaluation on wheat–*Th. intermedium* translocation lines

The wheat–*Th. intermedium* translocation lines Zhongke 78 and Zhongke 15 and the recurrent parent Jimai 22 were planted in rows with a length of 3 m and a spacing of 10 cm in a field in Beijing (E116°42′, N40°10′). Plant height was taken as an average of 10 randomly chosen tillers from five plants. For each line, the tillering number of 10 plants was counted. Ten spikes randomly selected from each line were used to calculate the spike length and kernel number per spike. The thousand kernel weight was measured three times by counting 500 kernels.

### Inoculation and the stripe rust resistance evaluation

The lines T14-44 and T14-42; the wheat–*Th. intermedium* translocation lines including Zhongke 15, Zhongke 353, Zhongke 215, Zhongke 16, Zhongke 55, and Zhongke 78; and the F_2_ individuals derived from Zhongke 15/Jimai 20 and Zhongke 78/Fielder were used for stripe rust resistance evaluation in a greenhouse. The wheat variety Mingxian 169 was used as the susceptible control. All plants were planted in the 32-cell trays in a greenhouse set at 16°C with a 16-h photoperiod. Fresh spores of three stripe rust races CYR32, CYR33, and CYR34 were independently suspended in distilled water. When the second leaf expanded, the seedlings were sprayed with the spore suspension. After inoculation, the seedlings were kept moist for 24 h at 12°C–13°C. Then, the plants were moved to a greenhouse set as 16°C with a 16-h photoperiod for approximately 14 days. Infection types were recorded based on a 0–4 scale (He et al., 2009). For the evaluation of adult plant resistance, the adult plant of Zhongke 78 and Zhongke 15 was evaluated in natural conditions in the field in Chengdu, Sichuan Province. The susceptible cultivar Mianyang 11 was used as control.

## Results

### TAI-14 karyotype and its stripe rust resistance

**Figure 1:**
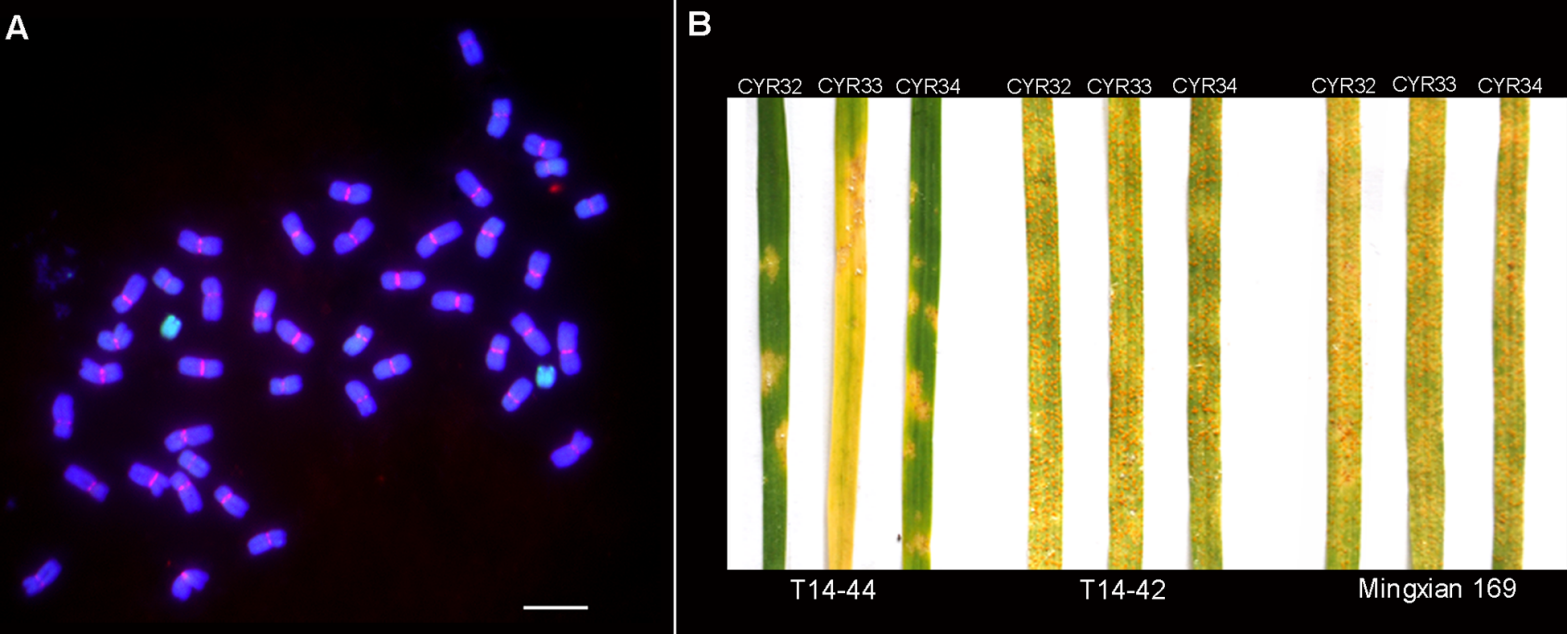
TAI-14 karyotype and its stripe rust resistance. **(A)** T14-44 karyotype identified by genomic *in situ* hybridization (GISH) and fluorescence *in situ* hybridization (FISH). The genomic DNA of *Th. intermedium* (green) and the wheat centromeric retrotransposon 6C6 (red) were used as a probe. The genomic DNA of Chinese Spring (CS) was used as a blocker. Chromosomes were stained with 4, 6-diamidino-2-phenylindole (DAPI; blue). Scale bar = 10 μm. **(B)** Stripe rust evaluation on T14-44 and T14-42. Stripe rust tests were performed using three rust races CYR32, CYR33, and CYR34, respectively. Common wheat cultivar Mingxian 169 was used as the susceptible control.

To characterize the karyotype of the wheat–*Th. intermedium* addition line TAI-14, we performed GISH and FISH analyses on the mitotic metaphase chromosomes. In addition to 42 chromosomes from wheat stained blue, a pair of chromosomes stained green was also detected in TAI-14, which indicated their origin of *Th. intermedium* (**Figure 1A**). Because of its smaller size and no detection of the CRW signal on it, the alien chromosome was classified as a telocentric chromosome (**Figure 1A**). In spite of having no CRW signals on the alien chromosome, functional centromeres were revealed by the presence of Centromere-specific histone H3 (CENH3) (Guo et al., 2016). However, we observed that the loss of the alien chromosome occurs to a certain extent in the self-crossing offspring, gradually transforming TAI-14 from T14-44 to T14-42. Using the isolates of races CYR32, CYR33, and CYR34, the evaluation on stripe rust resistance was performed on the seedlings of both T14-44 and T14-42. The addition line T14-44 showed resistance to all three races with an obvious hypersensitive response on the seedling leaves (**Figure 1B**). Conversely, the line T14-42 without an alien chromosome was susceptible to all three races and the vast numbers of fungal spores spread on the seedling leaves (**Figure 1B**). These results suggested that one genetic locus designated as *YrT14* on the alien chromosome was responsible for the stripe rust resistance. Further analysis discovered that the FISH marker St_2_-80 labeled the terminal region of the telocentric chromosomes in T14-44 (**Supplementary Figure 1**), which suggested that the alien chromosomes belong to the J or J^s^ subgenome rather than the St subgenome of *Th. intermedium*. Therefore, we found that one gene (*YrT14*) from the J or J^s^ subgenome of *Th. intermedium* conferred wheat with stripe rust resistance.

### Development and molecular cytogenetic analysis of wheat–*Th. intermedium* translocation lines

**Figure 2:**
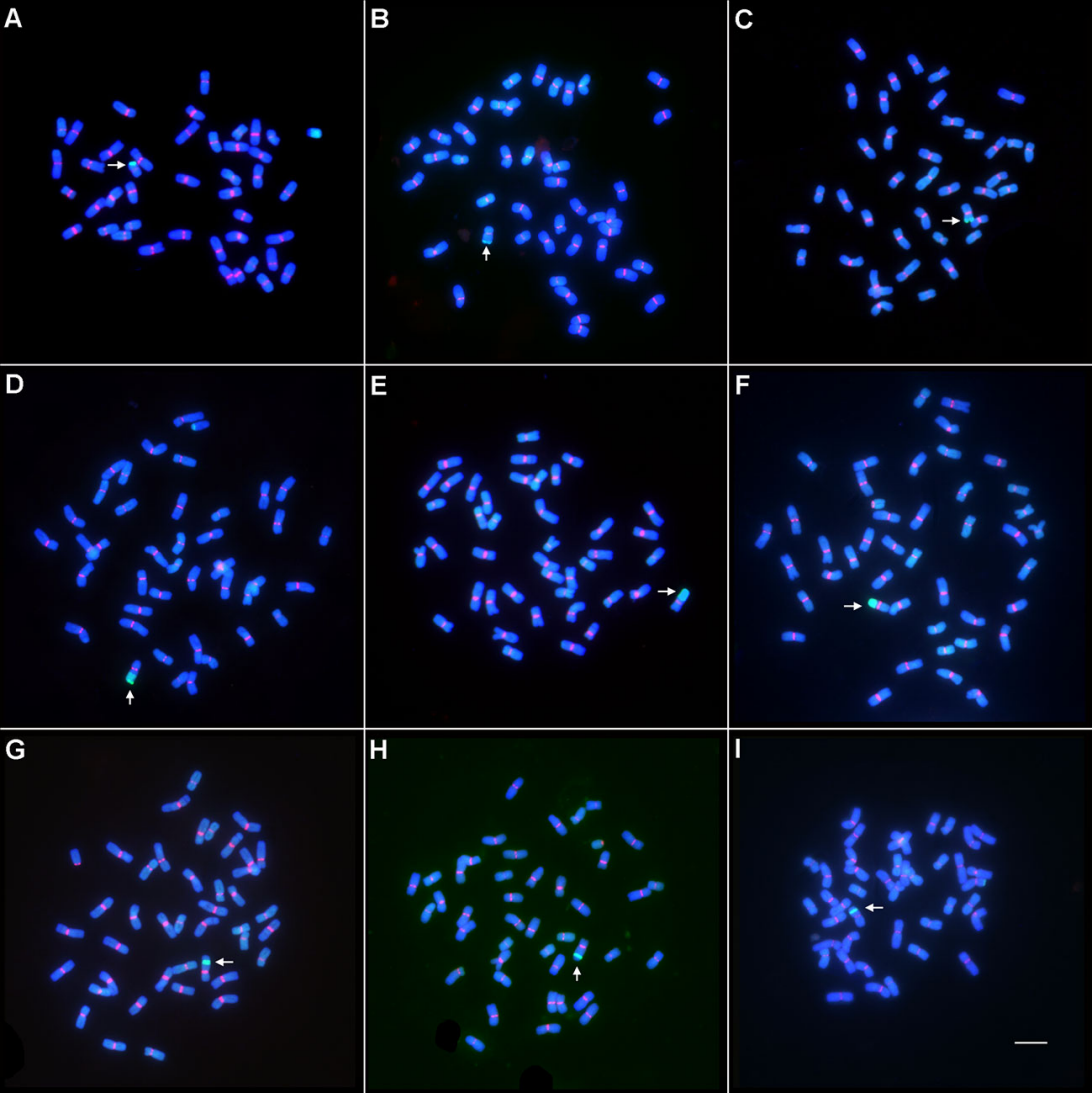
Wheat–*Th. intermedium* translocation line identified by GISH and FISH. The genomic DNA of *Th. intermedium* (green) was used as probe for GISH, and the wheat centromeric retrotransposon 6C6 (red) was used as probe for FISH. The genomic DNA of CS was used as a blocker. Chromosomes were stained with DAPI. Arrows indicated the translocated chromosomes. Scale bar = 10 μm. **(A–C)** The short alien segmental translocation lines identified by GISH and FISH. **(A)** Zhongke 55; **(B)** Zhongke 61; **(C)** Zhongke 316. **(D–F)** The long alien segmental translocation lines identified by GISH and FISH. **(D)** Zhongke 843; **(E)** Zhongke 70; **(F)** Zhongke 108. **(G–I)** The intercalary translocation lines identified by GISH and FISH. **(G)** Zhongke 21; **(H)** Zhongke 102; **(I)** Zhongke 127.

In order to transfer the alien resistance gene *YrT14* to common wheat, wheat–*Th. intermedium* translocation lines were produced by irradiating the pollen of TAI-14 at the flowering stage. The fresh pollens were then pollinated to the recurrent parent Jimai 22. By performing GISH on 8,100 F_1_ seeds, we obtained 153 wheat–*Th. intermedium* translocation lines at a frequency of 1.89% (**Table 1**). According to the size and position of the alien chromatin, the translocation lines were classified into three types (**Supplementary Figure 2**). If the ratio of the length of the alien fragment in translocation lines to the length of the telocentric chromosome ranged from 0 to 0.5, the translocation lines were classified as short alien segmental translocation lines, such as lines Zhongke 55, Zhongke 61, and Zhongke 316 (**Figures 2A–C**). If the ratio ranged from 0.5 to 1, they were classified as long alien segmental translocation lines, such as lines Zhongke 843, Zhongke 70, and Zhongke 108 (**Figures 2D–F**). If one part of the alien chromosome inserted into one wheat chromosome, the translocation lines were classified as intercalary translocation lines, such as lines Zhongke 21, Zhognke 102, and Zhongke 127 (**Figures 2G–I**). In detail, we obtained 66 short alien segmental translocation lines and 70 long alien segmental translocation lines, which accounted for 43.14% and 45.75%, respectively (**Table 1**). However, we only screened 17 (11.11%) intercalary translocation lines and the proportion was obviously lower than the other two types (**Table 1**).

**Table 1:** Three types of translocation lines identified from 8,100 seeds.

Translocation type	Number	Percentage (%)
Short alien segmental translocation line	66	43.14
Long alien segmental translocation line	70	45.15
Intercalary translocation line	17	11.11
Total	153	100

**Figure 3:**
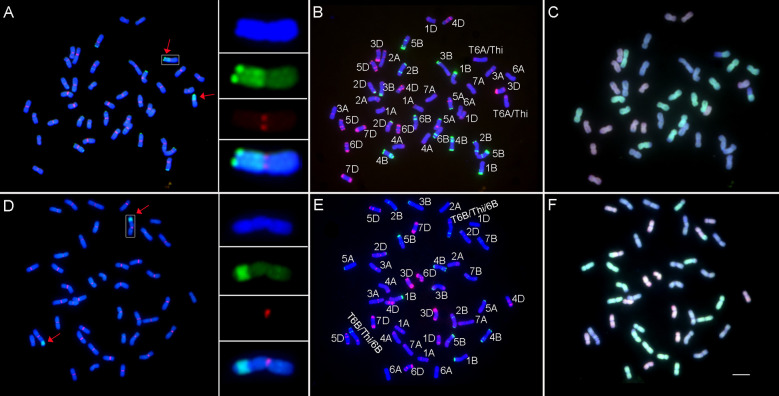
Cytological analysis on lines Zhongke 78 and Zhongke 15. **(A–C)** Cytological characterization of Zhongke 78. **(D–F)** Cytological characterization of Zhongke 15. GISH and FISH patterns of Zhongke 78 **(A)** and Zhongke 15 **(D)**. The genomic DNA of *Th. intermedium* (green) was used as probe for GISH and the wheat centromeric retrotransposon 6C6 (red) was used as probe for FISH. The genomic DNA of CS was used as a blocker. FISH patterns of Zhongke 78 **(B)** and Zhongke 15 **(E)** identified by Oligo-pTa535-1 (red) and Oligo-pSc119.2-1 (green). GISH patterns of Zhongke 78 **(C)** and Zhongke 15 **(F)** identified by the genomic DNA of *Aegilops tauschii* (DD) (red) and the genomic DNA of *Triticum urartu* (AA) (green). The genomic DNA of *Ae. speltoides* (SS) was used as a blocker. All chromosomes were counterstained with DAPI. Arrows indicated the translocated chromosomes. Scale bar = 10 μm.

To improve the integrated agronomic traits, all wheat–*Th. intermedium* translocation lines were backcrossed with Jimai 22 and homozygous translocation lines were selected among the offspring. Two homozygous translocation lines Zhongke 78 and Zhongke 15 with good agronomic traits were cytologically analyzed in detail (**Figure 3**). Zhongke 78 and Zhongke 15 were identified as a long segmental translocation line and an intercalary translocation line, respectively (**Figures 3A, D**). Combining multicolor GISH, the translocation in Zhongke 78 occurred on chromosome 6A and the alien chromatin nearly replaced the long arm of 6A (**Figures 3A–C**). For the line Zhongke 15, the inserted alien chromatin was identified on the chromosome 6B by using the probe Oligo-pSc119.2-1 (**Figures 3D, E**). In addition, the secondary constriction was observed near the inserted position proximal to the centromere (**Figure 3D**). Therefore, the alien chromatin was found inserted into the satellite of chromosome 6B in the line Zhongke 15.

Although both lines contained 42 chromosomes, the subgenomic components of Zhongke 78 were different from that of Zhongke 15 (**Figures 3C, F**). Upon further analysis, it was discovered that the number of all three subgenome chromosomes in Zhongke 15 was 14 (**Figure 3F**), whereas Zhongke 78 was found comprised of 16 A subgenome chromosomes, 12 B subgenome chromosomes, and 14 D subgenome chromosomes (**Figure 3C**). In detail, a pair of chromosomes 7B was absent and one more pair of intact chromosomes 6A was identified in Zhongke 78 (**Figure 3C**).

### Morphological evaluation and stripe rust resistance evaluation on Zhongke 15 and Zhongke 78

**Figure 4:**
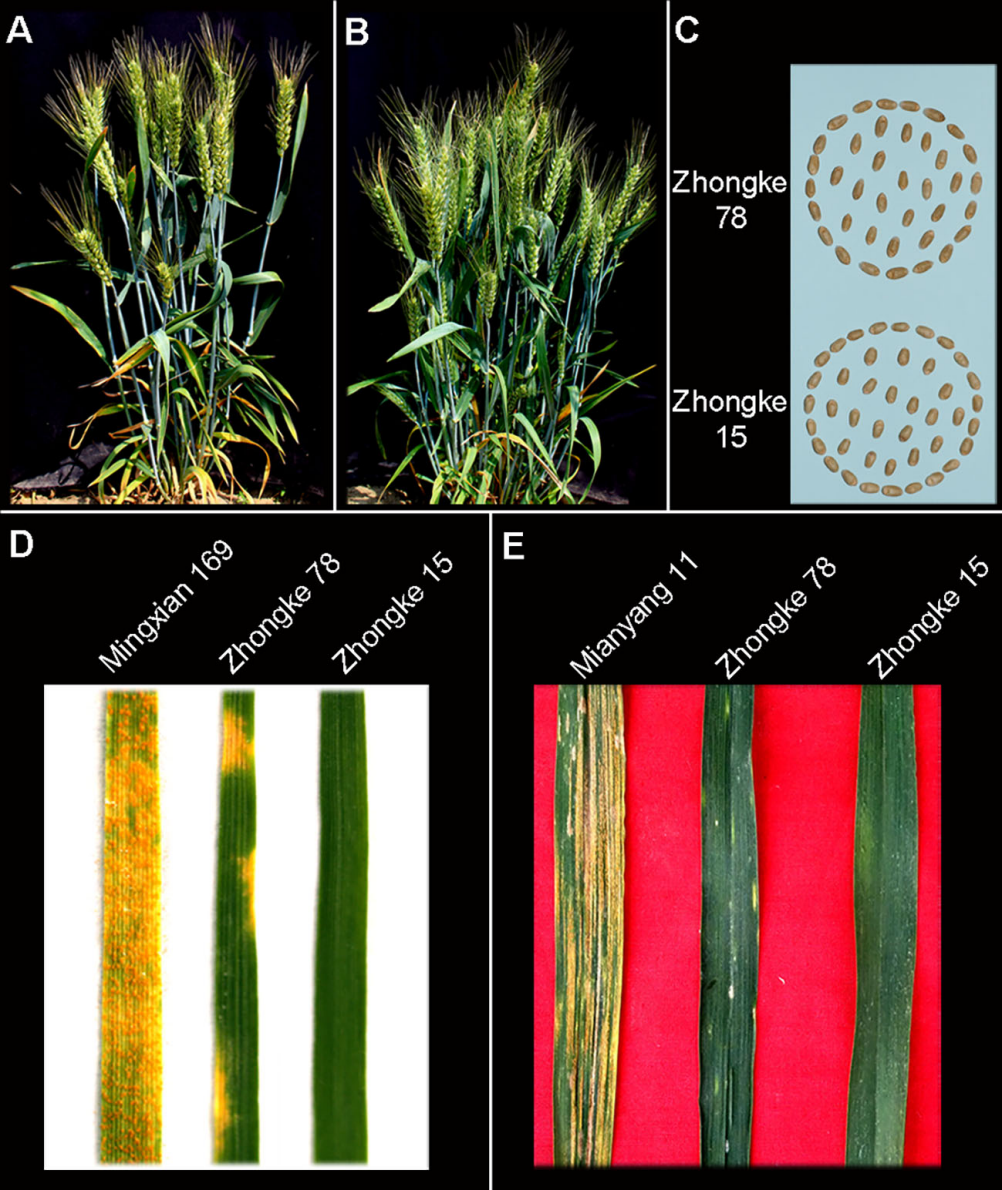
The integrated agronomic traits and stripe rust resistance of homozygous translocation lines Zhongke 78 and Zhongke 15. Phenotype of Zhongke 78 **(A)** and Zhongke 15 **(B)**. **(C)** Seeds of Zhongke 78 and Zhongke 15 harvested in the field. **(D)** Stripe rust resistance evaluation of Zhongke 78 and Zhongke 15 at the seedling stage in a greenhouse by mixed races CYR32 and CYR34. **(E)** Stripe rust resistance evaluation of Zhongke 78 and Zhongke 15 at the adult plant in the field of Chengdu, Sichuan province.

**Figure 5:**
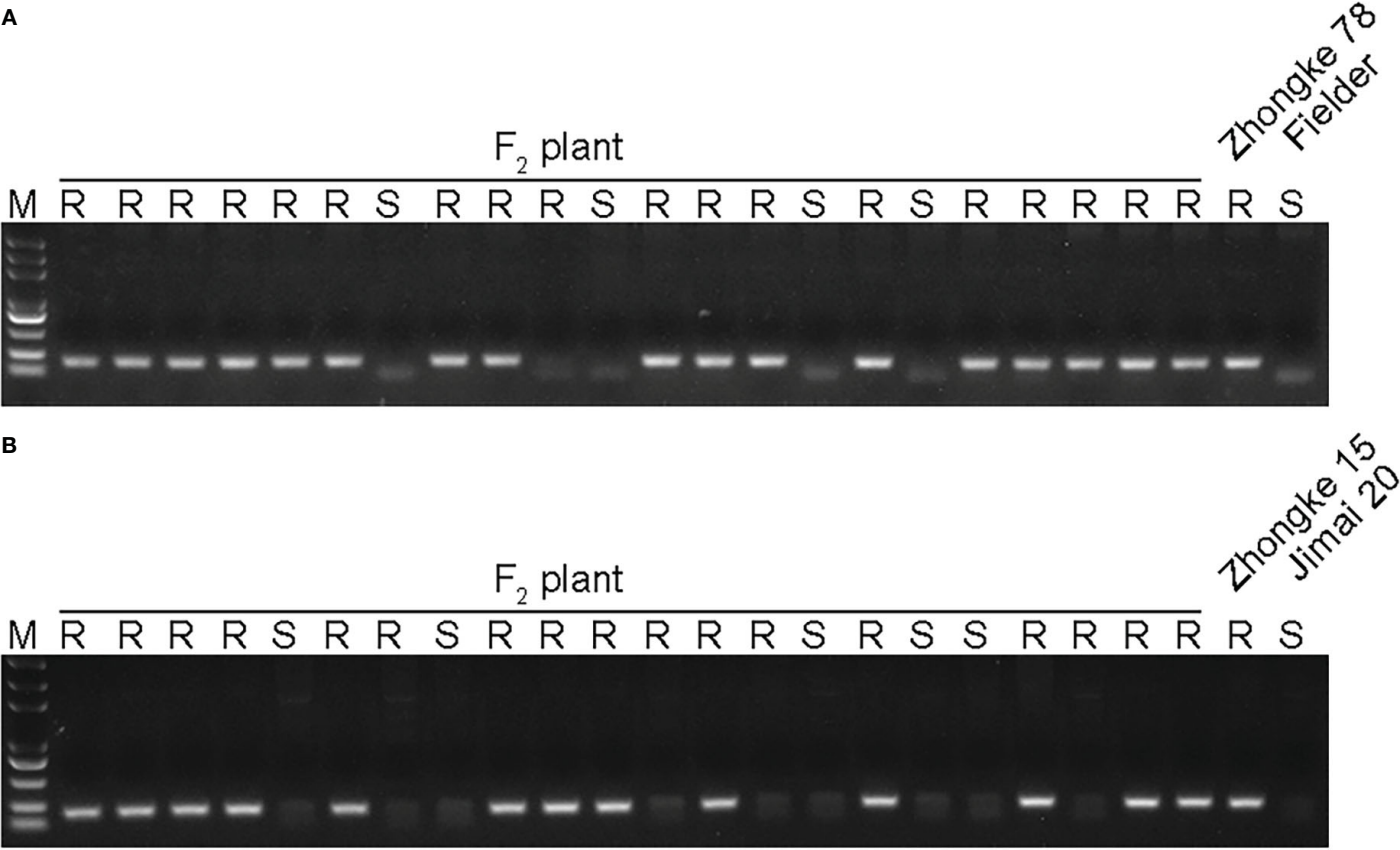
Detecting the alien chromatins among F_2_ plants derived from Zhongke 78/Field and Zhongke 15/Jimai 20 by using the specific marker T14K50. **(A)** Detecting the alien chromatin among F_2_ plants derived from the cross of Zhongke 78 and Fielder. **(B)** Detecting the alien chromatin among F_2_ plants derived from the cross of Zhongke 15 and Jimai 20. M, DNA marker. R, resistance to stripe rust. S, susceptible to stripe rust.

Both Zhongke 78 and Zhongke 15 were identified with many tillers, compact plant architecture, and filled seeds in morphology (**Figures 4A–C**, **Supplementary Figure 3A**). In detail, both two lines were higher in plant height and longer in spike length than their recurrent parent Jimai 22 (**Table 2**). Conversely, the kernel number per spike of both two lines were less than that of Jimai 22, with 26.2 of Zhongke 78 and 27.2 of Zhongke 15. In addition, we observed that the line Zhongke 15 exhibited more tillers but lower kernel weight than Jimai 22 while Zhongke 78 showed less tillers but a slightly higher kernel weight than Jimai 22 (**Table 2**). By inoculating the equally mixed isolates of races CYR32 and CYR34, both two lines were identified as resistant to stripe rust at the seedling stage in the greenhouse (**Figure 4D**). Furthermore, the adult plant stage resistance was also observed on both lines in the field of Chengdu, Sichuan Province (**Figure 4E**, **Supplementary Figures 3A, B**). In order to exclude the effect of the recurrent parent Jimai 22 on the stripe rust resistance, two susceptible cultivars, Fielder and Jimai 20, were employed to produce F_2_ segregation populations. We performed stripe rust resistance on 218 F_2_ progenies derived from the cross of Zhongke 78 and Fielder and 225 progenies derived from the cross of Zhongke 15 and Jimai 20 using the isolate of race CYR34. We identified 186 resistant progenies and 32 susceptible progenies from the segregation population derived from Zhongke 78 and Fielder. Similarly, among the F_2_ progenies derived from Zhongke 15 and Jimai 20, 208 progenies were identified as resistant and 17 were identified as susceptible (**Table 3**, **Supplementary Figure 4**). To facilitate the association analysis between stripe rust resistance and alien chromatin, the specific marker T14K50 was used to track the alien chromatins that derived from Zhongke 78 and Zhongke 15 (**Figure 5**). We found that the marker T14K50 can be only detected in the resistant progenies but not in the susceptible ones for both F_2_ segregation populations (**Table 3**, **Figure 5**). In other words, all F_2_ progenies carrying the translocated chromosome derived from Zhongke 78 and Zongke 15 were resistant to stripe rust and the translocated chromosome cannot be detected in all susceptible ones. Therefore, the stripe rust resistance was cosegregated with the alien chromatins in Zhongke 78 and Zhongke 15, which confirmed that the stripe rust resistance originated from *Th. intermedium*.

**Table 2:** Phenotypic analysis on wheat–*Th. intermedium* translocation line Zhongke 78 and Zhongke 15.

Trait	Zhongke 78		Zhongke 15		Jimai 22	
	Mean	Range	Mean	Range	Mean	Range
Plant height (cm)	91.1^c^	89.3–92.5	81.1^b^	79.4–82.6	80.0^a^	79.2–80.6
Tillering number	8.9^a^	7–12	11.8^b^	9–18	9.1^a^	8–11
Spike length (cm)	9.8^c^	9.1–10.4	9.3^b^	8.9–9.5	8.6^a^	8.4–8.8
Kernel number per spike	26.2^b^	25–28	27.2^b^	25–29	28.4^a^	27–30
Thousand kernel weight (g)	42.3^a^	42.1–42.5	40.6^b^	40.5–40.8	42.2^a^	42.1–42.2

Means followed by different letters within a column were significantly different at P < 0.05.

**Table 3:** Stripe rust resistance test and genotyping on two F_2_ populations.

Cross	Resistant Progeny		Susceptible progeny		Total
	T14K50/+	T14K50/-	T14K50/+	T14K50/-	
Zhongke 78/Fielder	135	51	0	32	218
Zhongke 15/Jimai 20	146	62	0	17	225

### Development of markers for the alien chromosome and physical mapping of *YrT14*

**Figure 6:**
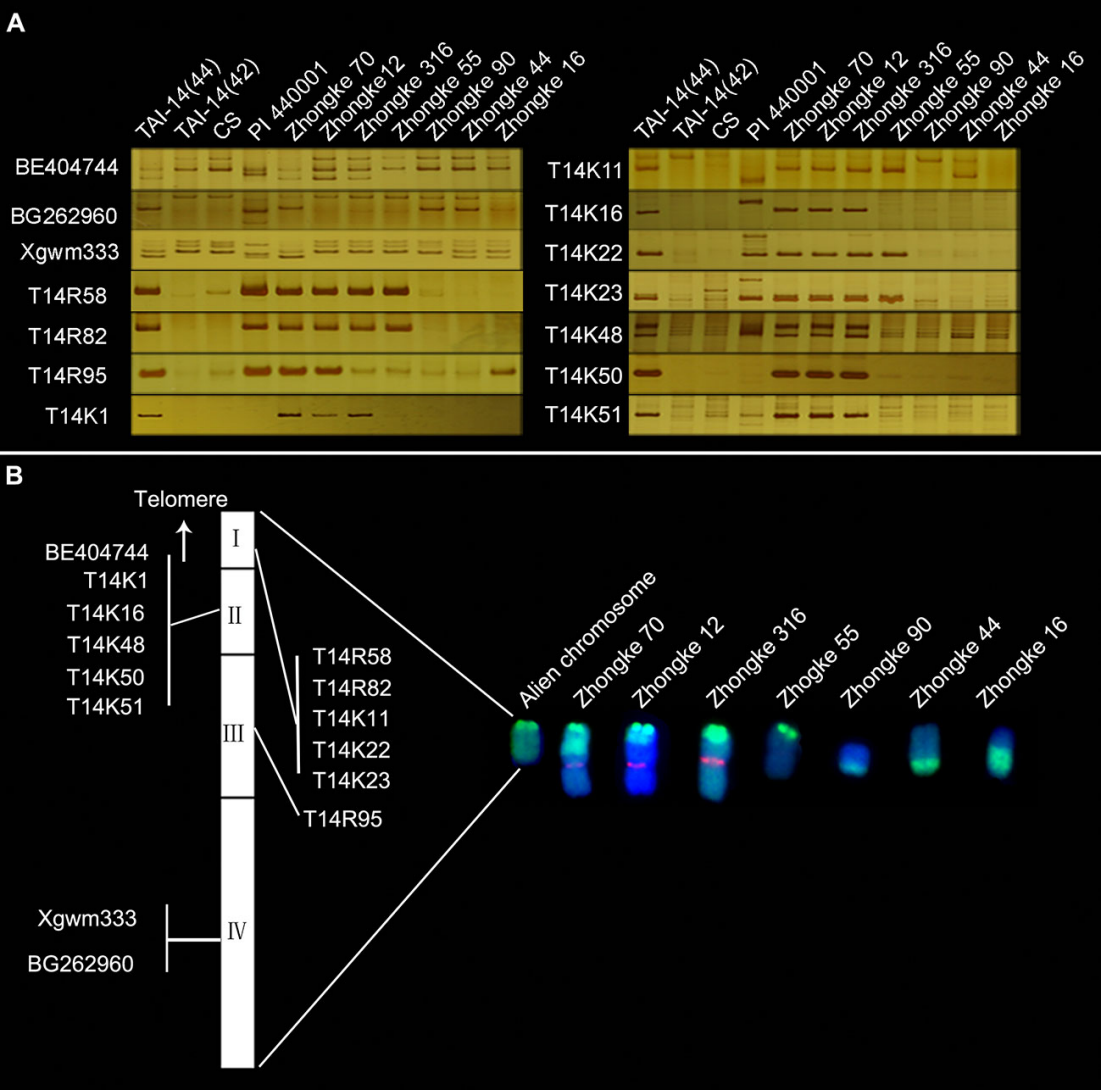
Specific markers and a physical map for the alien chromosome. **(A)** Specific markers for the alien chromosome and their distribution on seven translocation lines with different patterns. Common wheat CS and the *Th. intermedium* accession PI 440001 were used as control lines for exploiting specific markers for the alien chromosome in TAI-14. **(B)** Physical map construction for the alien chromosome.

**Figure 7:**
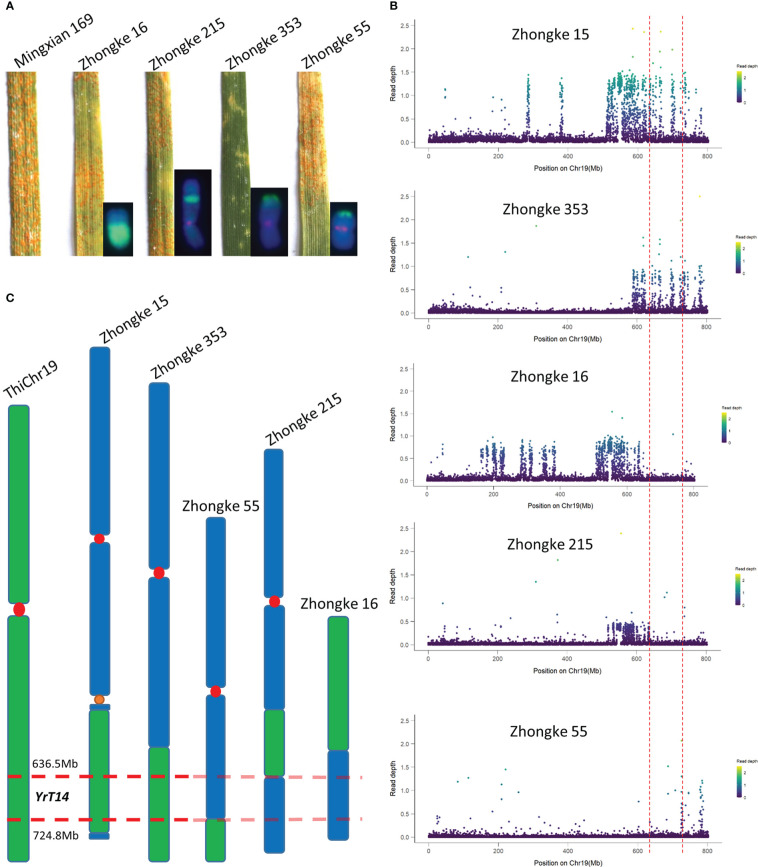
The physical mapping of the stripe rust resistance gene *YrT14*. **(A)** Stripe rust resistance evaluation on the wheat–*Th. intermedium* translocation lines Zhongke 16, Zhongke 215, Zhongke 353 and Zhongke 55. **(B)** Genome resequencing and the read depth analysis on Zhongke 15, Zhongke 353, Zhongke 16, Zhongke 215, and Zhongke 55. **(C)** Mapping *YrT14* by relying on the overlaps among different wheat–*Th. intermedium* lines.

To develop specific markers for the alien chromosome to facilitate gene mapping, 197 wheat SSR and 88 EST markers from wheat group 7 were firstly screened. However, only three specific markers for the alien chromosome were obtained, including one SSR marker Xgwm333 and two EST markers BE404744 and BG262960 (**Table 4**, **Figure 6A**). In order to obtain more markers for the alien chromosome, we secondly annotated 2,632 *de novo* transcripts that only expressed in T14-44 but not in T14-42. Among them, 39 transcripts annotated as NBS-LRR protein and 94 transcripts annotated as protein kinase were used to design primers. At last, three markers from the NBS-LRR transcripts and eight markers from the protein kinase transcripts were developed (**Table 4**, **Figure 6A**). Totally, we developed 14 specific markers for the alien telocentric chromosome in TAI-14. In addition, we checked the distribution of all markers on seven translocation lines with different translocated patterns by polyacrylamide gel electrophoresis (**Figure 6A**). Finally, the alien chromosome was divided into four parts and a physical map was constructed with each part anchoring one-to-six markers (**Figure 6B**). To map the resistance gene *YrT14*, three wheat–*Th. Intermedium* lines that were susceptible to stripe rust were selected to exclude the intervals without *YrT14* (**Figure 7A**). The stripe rust–susceptible line Zhongke 16 was proven to carry a large alien chromatin close to the centromere and including regions III and IV of the physical map revealed by molecular markers (**Figures 6**, **7A**). Unlike Zhongke 16, the stripe rust–susceptible line Zhongke 55 was proven to carry a small alien chromatin close to the telomere and only including the regions I of the physical map (**Figures 6**, **7A**). Therefore, based on these results, we roughly mapped the resistance gene *YrT14* to the region II of the physical map (**Figure 6B**).

**Table 4:** Specific markers and primer sequences for the alien telocentric chromosome.

Primer^a^	Sequence (5´-3´)	Tm (°C)	Type
Xgwm333F	GCCCGGTCATGTAAA ACG	55	SSR
Xgwm333R	TTTCAGTTTGCGTTAAGCTTTG		
BE404744F	TGGGATGAGTTGCTTGACA	60	EST
BE404744R	GCGTTGACGATGACTTGTGT		
BG262960F	AAGTGGATGAGAAGCCGTTT	60	EST
BG262960R	CTGAGCTGAGCAGCACATAA		
T14K1F	GTTGCGCAGACGTGACAATA	58	RNA-Seq
T14K1R	ACACGAACTGTGACGGCTAT		
T14K11F	ATCGGCGTAATCTTCCCGAC	58	RNA-Seq
T14K11R	ATGTCAAAAGGCCTGGGGAG		
T14K16F	TGTTGCCGTCTACCAGCTTT	58	RNA-Seq
T14K16R	GAGCGTTGACCAGCACCATA		
T14K22F	GTCTCTTCTCGTCGAGGCAC	58	RNA-Seq
T14K22R	CCCTCGGATTACACAGCGAA		
T14K23F	TACGGTGCACTGACAGAGTC	58	RNA-Seq
T14K23R	GAGCCTGGGCAGAGTGATTT		
T14K48F	GGAAAGATGTGGCATCACGG	58	RNA-Seq
T14K48R	GCAGTGGGTTGCATTTCCTT		
T14K50F	CTCCGAAGTACTCCCTGTGG	58	RNA-Seq
T14K50R	TCCACCGATTTGATACTCGGC		
T14K51F	AGATGTGTCATCACGGTGGA	58	RNA-Seq
T14K51R	GCGGGGACATGGTAAACTACA		
T14R58F	CCCTTCAACCGGAGGGATATG	58	RNA-Seq
T14R58R	TATTGCCCAGAGTCGTCCCA		
T14R82F	TGACCAGTTGTGATGGGACG	58	RNA-Seq
T14R82R	AGGTCTTTATTGCCCGCGAA		
T14R95F	AGACAACGGCTGCTCGGTAA	58	RNA-Seq
T14R95R	TCAAGGGTAATGTGGTCGCC		

a The marker names used in this study.

To further narrow down the candidate interval of *YrT14*, five wheat–*Th. intermedium* translocation lines including the said two stripe rust–susceptible ones as well as Zhongke 215, Zhongke 353, and Zhongke 15 were selected to perform genome resequencing. Compared with the line Zhongke 15, Zhongke 215 was also identified as an intercalary translocation line but susceptible to stripe rust (**Figure 7A**). Except for Zhongke 15 and Zhongke 78, the translocation line Zhongke 353 was also identified as resistant to stripe rust and confirmed by the association analysis between stripe rust and the alien chromatin (**Figure 7A**, **Table S2**). After mapping the reads to the reference genome, we found that the alien chromatin in our developed translocation lines was corresponding to the chromosome 19 (Chr19) of the assembled reference genome of *Th. intermedium* (**Figure 7B**). Read depth analysis revealed that the stripe rust–resistant lines Zhongke 15 and Zhongke 353 shared a common interval ranging from 586.9 to 782.0 Mb (**Figure 7B**). Based on the results of the stripe rust–susceptible line Zhongke 55, we excluded the intervals from 724.8 Mb to the telomere at the end of the alien chromosome (**Figure 7B**). Similarly, the interval from the centromere to 636.7 Mb was also excluded according to the results of the stripe rust–resistant lines Zhongke 16 and Zhongke 215 (**Figure 7B**). Therefore, relying on the overlap among the five above-mentioned translocation lines, the stripe rust resistance gene *YrT14* was narrowed down to an approximately 88.1 Mb interval from 636.7 to 724.8 Mb on *Th. intermedium* Chr19 corresponding to chromosome 7J or 7J^s^ (**Figure 7C**, **Supplementary Figure 1**).

## Discussion

### Exploiting a novel stripe rust resistance gene from *Th. intermedium* for wheat improvement

Exploiting genes from wild relatives is an important way for wheat improvement. As a wild relative species containing many beneficial genes for both biotic and abiotic stresses, *Th. intermedium* has been widely used in wheat improvement because of its crossability with wheat. It was reported that several stripe rust resistance genes have been mapped on the different St chromosomes of *Th. intermedium*, including chromosomes 1St, 2St, 3St, and 7St (Hu et al., 2011; Song et al., 2013; Nie et al., 2019; Wang et al., 2022). Except for the St subgenome, the subgenome J or J^s^ has also been proven to carry the resistance genes for stripe rust. A gene for stripe rust resistance in the wheat–*Th. intermedium* addition line Z4 was located to the short arm or in the proximal region of the long arm of chromosome 7J^s^ (Lang et al., 2018). Two other stripe rust resistance genes *Yr50* and *YrL693* were reported in wheat–*Th. intermedium* introgression lines CH223 and L693, respectively. However, both genes were putatively derived from *Th. intermedium* based on molecular mapping while the *Th. intermedium* chromatin cannot be detected on wheat chromosomes (Liu et al., 2013; Huang et al., 2014). In this study, the genetic analysis revealed the location of a resistance gene for stripe rust on the alien chromosome in the wheat–*Th. intermedium* addition line TAI-14. It was demonstrated that the alien chromosome in TAI-14 was homoeologous with wheat group-7 chromosomes (Gao and Hao, 1993). This was confirmed in our study by the fact that three markers from the long arms of wheat group-7 chromosomes, Xgwm333, BE404744, and BG262960, were identified specific for the alien chromosome. We demonstrated that the alien chromosome in TAI-14 did not belong to the St subgenome by using the FISH marker St_2_-80 reported by Wang et al. (2017). In addition, the genome resequencing data supported that the alien telocentric chromosome was corresponding to the long arm of Chr19. More importantly, the resistance gene *YrT14* was located to an interval ranging from 636.5 to 724.8 Mb, which was far away from the short arm and the proximal region of the long arm reported by Lang et al. (2018). Therefore, in this study, we demonstrated that the long arm of chromosome 7J or 7J^s^ from *Th. intermedium* was also responsible for stripe rust resistance and the gene *YrT14* on it was a novel gene that was not reported before.

### New wheat–*Th. intermedium* translocation lines for wheat stripe rust—resistant breeding

Translocation lines made it possible to transfer useful genes from related species to wheat. Linkage drag is a key factor affecting the success of the alien gene transfer (Faris et al., 2008). No differences on agronomic traits were observed between T14-44 and T14-42 in the field (**Supplementary Figure 5**), which indicated that the alien telocentric chromosome did not carry genes that have deleterious effects on yield and quality. However, the loss of the alien chromosome in TAI-14 occurred to a certain extent in the self-crossing offspring, gradually transforming TAI-14 from T14-44 to T14-42 and finally resulting in losing resistance to stripe rust. Therefore, in this study, we chose to transfer the stripe rust-resistance gene *YrT14* to common wheat by the mean of the translocation line.

Physical radiation can break both wheat and wild relative chromosomes at a different position in addition lines. It was reported that classic Non-homologous end joining (NHEJ) junctions produced translocation lines during Double strand break (DSB) repair (Gillert et al., 1999). We obtained 153 wheat–*Th. intermedium* translocation lines by irradiating the pollen of the addition line TAI-14, including 66 short alien segmental translocation lines, 70 long alien segmental translocation lines, and 17 intercalary translocation lines. Up to date, two wheat–*Th. intermedium* translocation lines Zhongke 15 and Zhongke 78 with good integrated agronomic traits have been selected from the backcrossing offspring. We employed F_2_ populations to exclude the effect of the recurrent parent Jimai 22 on the resistance to stripe rust and confirmed that the alien chromatins in both Zhongke 15 and Zhongke 78 were cosegregated with the stripe rust resistance.

China is one of the largest stripe rust epidemic regions in the world (Stubbs, 1988; Wan et al., 2004). It was reported that the CYR32 and CYR33 races were accounted the highest frequencies among all tested races from 1997 to 2007 in China because of their parasitic fitness on leading commercial cultivars (Wan et al., 2007; Wang et al., 2009). The appearance of CYR34 has overcome the resistance of many wheat cultivars with *Yr26* located on chromosome 1BS (Ma et al., 2001; Wang et al., 2008; Han et al., 2015). It was predicted to develop into a new predominant race and may cause stripe rust epidemics in China (Bai et al., 2018; Wang et al., 2019). Therefore, it is of great importance to exploit new resistant sources that can protect wheat from races CYR32, CYR33, and CYR34. In our study, the gene *YrT14* was identified highly resistant to all three races. Therefore, Zhongke 15 and Zhongke 78 should be incorporated into the pool of germplasm as novel valuable resources for wheat stripe rust–resistant breeding.

### Intercalary translocation lines with alien chromatin inserting into wheat satellite

The usefulness of the translocation lines depended on whether the alien fragment can compensate for the replaced wheat segments as well as the linkage drag (Faris et al., 2008). As the alien chromosome in TAI-14 was homoeologous to the wheat group-7 chromosome, it may compensate the loss of 7B chromosome to some extent in Zhongke 78. Except for compensated translocation lines, the intercalary translocation line with an alien chromatin carrying targeted genes and no deleterious genes inserting into a wheat chromosome and without the loss of any wheat chromatin would be an ideal type. A wheat–*Agropyron cristatum* intercalary translocation line Pubing 3035 was reported to enhance thousand-grain weight and spike length in two populations (Zhang et al., 2015). In our study, Zhongke 15 was identified as an intercalary translocation line without the loss of any wheat chromosome. More interestingly, we found that a segment of the alien chromatin was inserted into the satellite of chromosome 6B, making the satellite much bigger than before. Several intercalary translocation lines between wheat and wild relatives have been reported (Mukai et al., 1993; Chen et al., 2008; Luan et al., 2010; <xr rid="r8">Chen et al., 2012</xr>). However, this is the first time to our knowledge that an intercalary translocation into the satellite region has been reported.

In China, the 1BL/1RS translocation lines were introduced from Europe and Russia and widely used in wheat breeding because of their stripe rust and powdery mildew resistance. Several important genes were found located on the satellite of chromosome 1RS, such as the resistance genes against powdery mildew (*Pm8*), stripe rust (*Yr9*), leaf rust (*Lr26*), and stem rust (*Sr31*) (Mago et al., 2005; Sharma et al., 2011). To a certain extent, the newly formed satellite with *YrT14* in Zhongke 15 is similar to the satellite of chromosome 1R. All these suggested that the intercalary translocation line Zhongke 15 had great potential for wheat stripe rust resistance breeding.

### Physical mapping and clone of *YrT14*

GISH and FISH are two powerful cytogenetic techniques for identifying alien chromatin on a wheat background. However, they cannot distinguish the accurate size of the alien fragment at the molecular level. In this study, by resequencing five wheat–*Th. intermedium* translocation lines and relying on their overlap, we mapped the stripe rust resistance gene *YrT14* to an approximately 88.1 Mb interval ranging from 636.7 to 724.8 Mb on *Th. intermedium* Chr19. Although the candidate interval was narrowed down, it was still too large to exploit the gene *YrT14*. To resolve the issues, on the one hand, we could create a mutant to produce genetic populations to fine-map the resistance gene *YrT14*. On the other hand, with the insertion of the alien chromatin, the translocated chromosome T6B/Thi/6B in the line Zhongke 15 was obviously larger in size than other chromosomes, which opened up the possibility of using mutants in conjunction with chromosome sorting and sequencing to facilitate the cloning of the candidate gene (Dolezel et al., 2014; Sánchez-Martín et al., 2016). In addition, the read depth analysis in this study revealed great differences between the alien chromatin in TAI-14 and Chr19 in the reference genome, which might be caused by the difference between *Th. intermedium* accessions and the incomplete reference genome. Therefore, the stripe rust–susceptible mutant and a more complete reference genome of *Th. intermedium* are the prerequisites for the cloning of *YrT14*.

## Conclusions

In this study, the wheat–*Th. intermedium* addition line TAI-14 was identified as resistant to three dominant stripe rust races CYR32, CYR33, and CYR34. Furthermore, we demonstrated the presence of a novel resistance gene (*YrT14*) for stripe rust on the alien chromosome. To transfer the resistance gene to common wheat, γ irradiation was performed on the line TAI-14. A great number of wheat–*Th. intermedium* translocation lines were developed, including 66 short alien segmental translocation lines, 70 long alien segmental translocation lines, and 17 intercalary translocation lines. Among them, the long alien segmental translocation line Zhongke 78 and the intercalary translocation line Zhongke 15 were identified as resistant to stripe rust and showing good integrated agronomic traits. Cytological analysis revealed that the alien chromatin nearly replaced the long arm of chromosome 6A in Zhongke 78, while a segment of the alien chromatin was inserted into the satellite of chromosome 6B in Zhongke 15. Both lines could be used as novel resources for wheat stripe rust resistance breeding. In addition, by screening SSR and EST markers as well as the markers developed from RNA-Seq data, 14 specific markers were identified for the alien chromosome and a physical map was constructed. The linked marker T14K50 can be used for molecular marker–assisted breeding. Finally, based on the karyotype, reaction to stripe rust, and genome resequencing data of different wheat–*Th. intermedium* translocation lines, the stripe rust resistance gene *YrT14* was located to an 88.1 Mb interval ranging from 636.7 to 724.8 Mb on *Th. intermedium* chromosome 19.

## Material availability statement

The wheat–*Th. intermedium* translocation lines developed in this study were available from the corresponding author to be required for academic study.

## Data availability statement

The raw sequencing data was deposited in NCBI database under number PRJNA911033. All other data are included in the article.

## Author contributions

XG, WY, and FH planned and designed the research. XG, YH, JW, SF, CW, MW, CZ, XH, and TW performed experiments, conducted fieldwork, and performed evaluation on stripe rust resistance. XG and FH wrote the manuscript with contributions from all authors. All authors contributed to the article and approved the submitted version.
