# Supplementary figures and images for: Development and cytological characterization of wheat–Thinopyrum intermedium translocation lines with novel stripe rust resistance gene
Source: Front Plant Sci. 2023 Feb 24;14:1135321. doi: 10.3389/fpls.2023.1135321 (PMC9998693; doi:10.3389/fpls.2023.1135321)

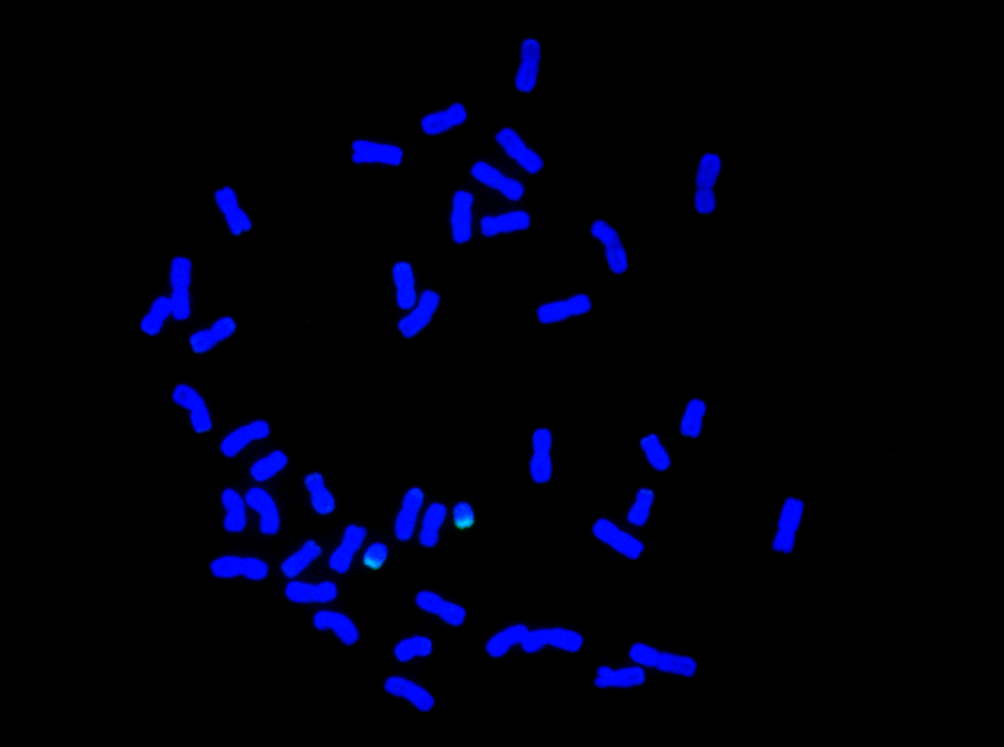

Supplement: Supplementary file 1 [file Image_1.tif]

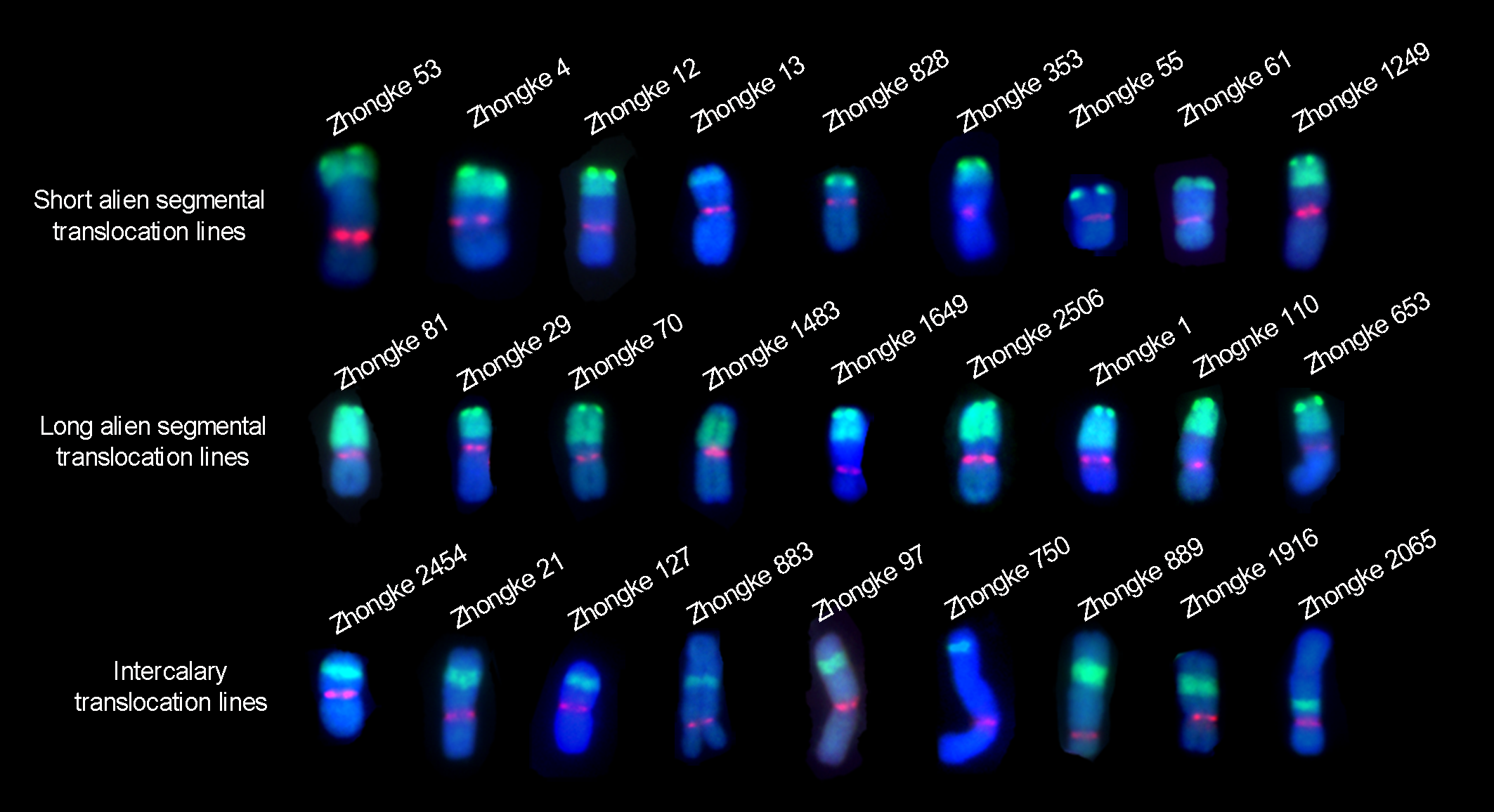

Supplement: Supplementary file 2 [file Image_2.tif]

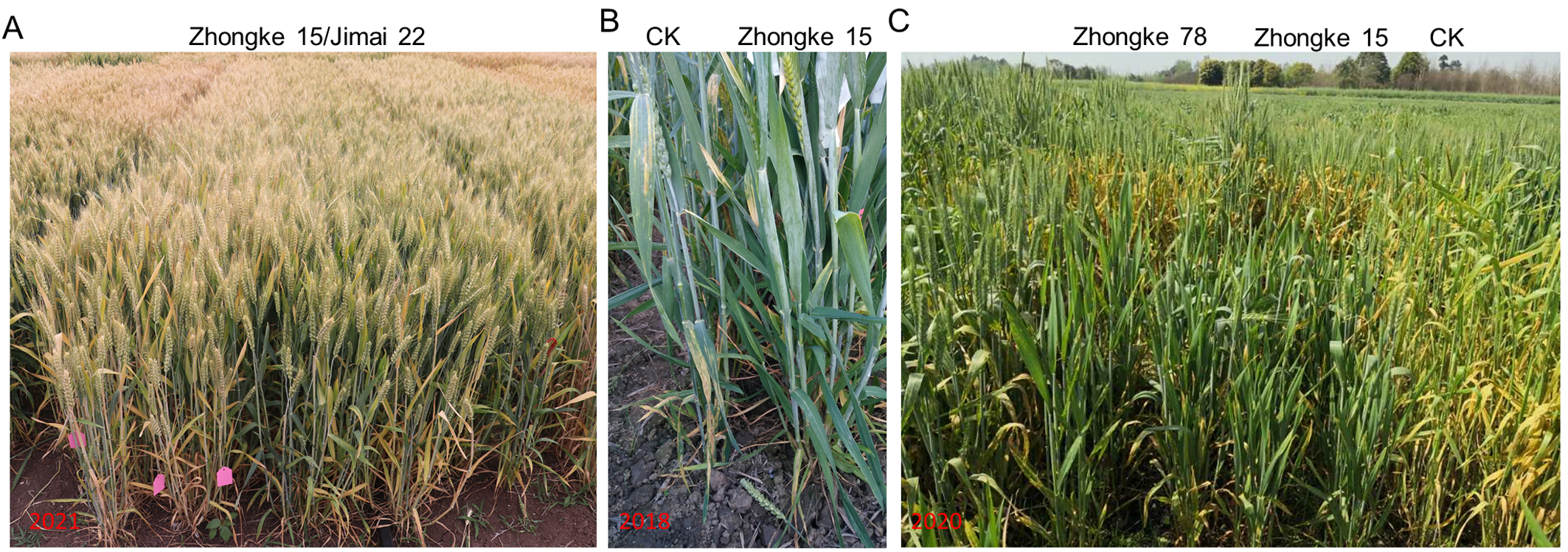

Supplement: Supplementary file 3 [file Image_3.tif]

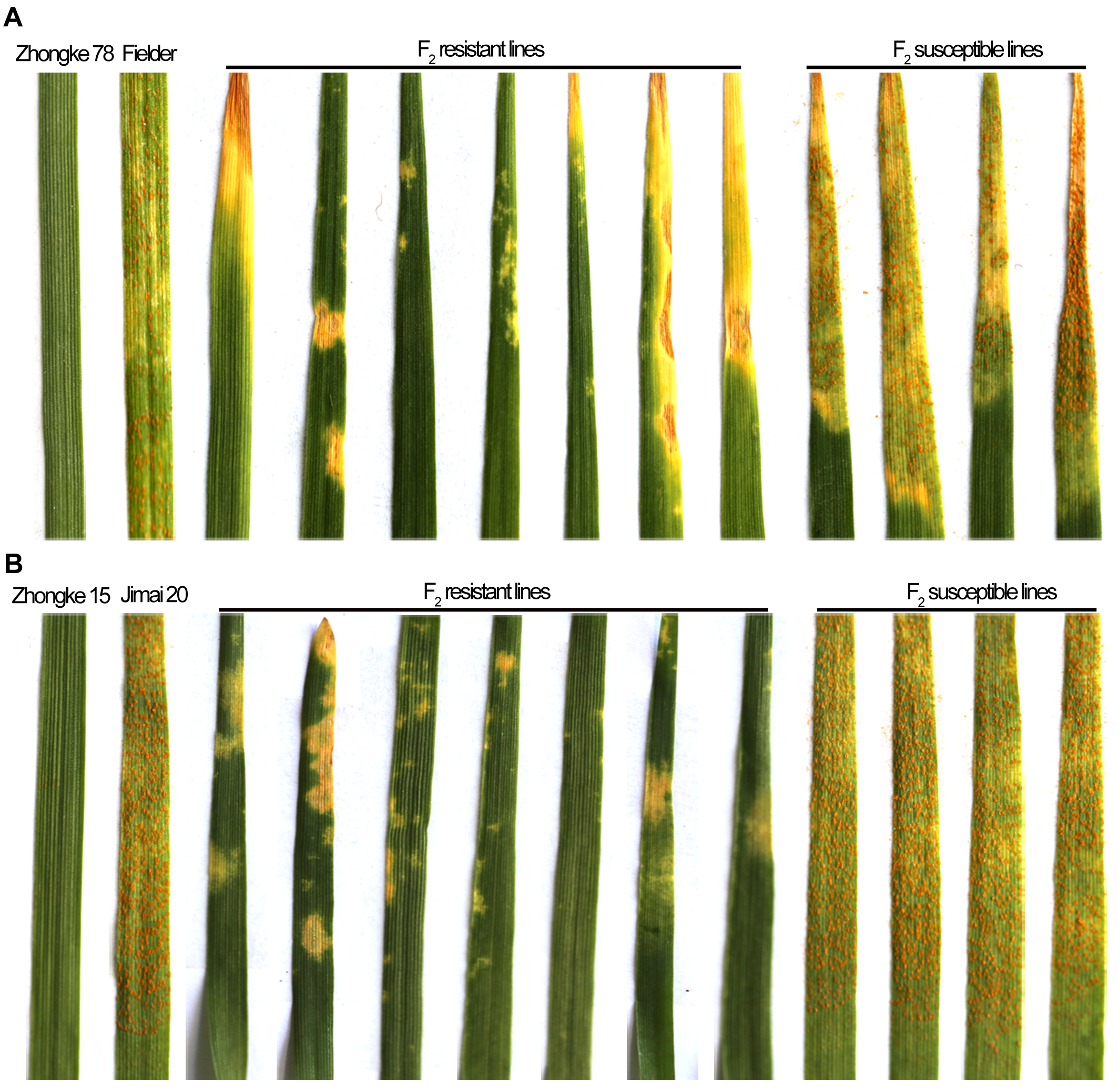

Supplement: Supplementary file 4 [file Image_4.tif]

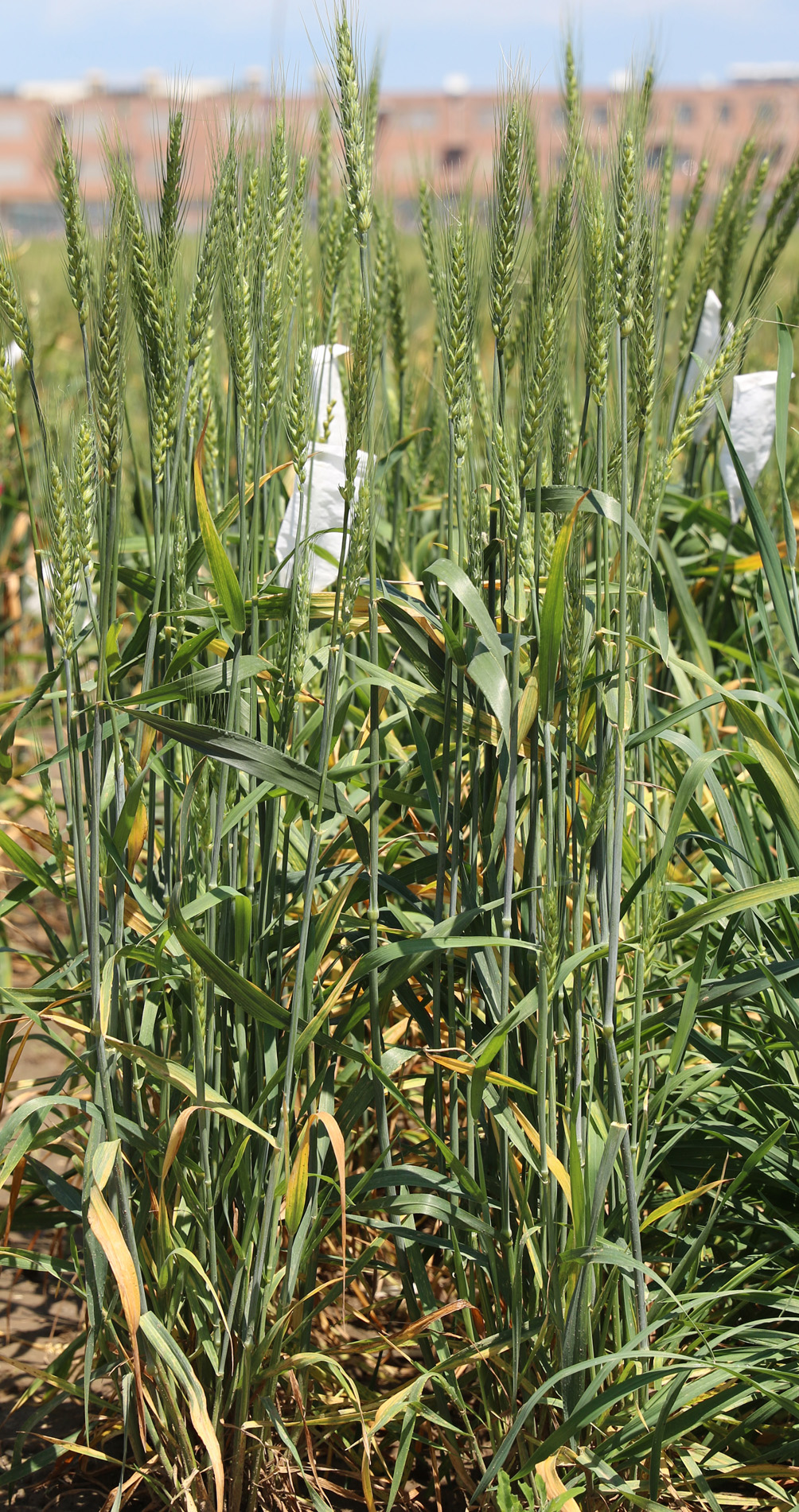

Supplement: Supplementary file 5 [file Image_5.jpeg]
